# The Relationship Between Gender Identity, Economic Stressors, Social Support, Concurrent Substance Use and Suicidal Ideation

**DOI:** 10.21203/rs.3.rs-4618444/v1

**Published:** 2024-07-19

**Authors:** Sara Kelly, Sarah Donohue, Kathleen Rospenda, Kristin Moilanen, Niranjan Karnik, Jesse Herron, Timothy Johnson, Judy Richman

**Affiliations:** University of Illinois College of Medicine Peoria; University of Illinois College of Medicine Peoria; University of Illinois at Chicago; University of Illinois at Chicago; University of Illinois at Chicago; University of Illinois at Chicago; University of Illinois at Chicago; University of Illinois at Chicago

## Abstract

**Purpose:**

To examine a comprehensive list of demographic, substance use, economic, and social factors associated with suicidal ideation (SI) among middle-aged adults.

**Methods:**

Cross-sectional data were obtained from a national sample of middle-aged adults between February and November 2022. The study’s final sample include 1,337 respondents who represented the adult population of persons aged 40–60 years in the United States. Bivariate and multivariate statistics were employed to identify significant factors associated with past year SI, in particular single vs. multiple instances of SI.

**Results:**

Of the sample, 140 (10.4%) reported SI in the past year. Among those, more than half (60.0%, n = 84) reported SI multiple times in the past year. Multivariable logistic regression indicated that those who were a gender minority, engaged in concurrent substance use, or had financial stressors had significantly higher odds of past SI. Multinomial regression found that concurrent substance use (adjusted odds ratio [aOR] 3.17; 95% confidence interval [CI] 1.76–5.70) and having a lower standard of living than their parents/caregivers (aOR 2.99; 95% CI 1.39–6.41) predicted repeated past year SI whereas higher social support was protective against multiple SI experiences (aOR 0.65; 95% CI 0.55–0.78).

**Conclusion:**

Gender minorities and those reporting concurrent substance use had the highest odds of past year SI. Findings underscore the need to develop public health and clinical interventions tailored to these highest-risk middle-aged adults in order to prevent suicide.

## Background

Suicide and suicidality continue to be a serious public health crisis with growing mortality rates throughout the United States (US) [1–3]. Over the past decade, suicide mortality rates increased by 16.3% in the US, from 12.3 to 14.3 per 100,000 between 2011 and 2021 [4]. However, suicide rates are not homogenous across population subgroups. Suicide among middle-aged adults, in particular, is a growing public health problem as adults aged 35–64 years account for 46.8% of all suicides in the US and it is the eighth leading cause of death for this age group [4–5]. Middle-aged adults, specifically those aged 45 to 54 years old, had the highest increase in mortality rate from 2011 to 2021, with a suicide mortality rate of 12.7 in 2011 and 18.2 per 100,000 in 2021. This equates to a 43.3% increase in mortality rate, more than double the rate of change across the general population [4].

Given the growing concern over this issue in the United States, millions of dollars in funding have been distributed to suicide prevention programs [5]. However, there is no one-size fits all intervention that works [6–7]. Despite numerous resources allocated to reduce suicide rates, other indicators of mental health distress, such as substance use and overdose, have been on the rise as well. As outlined by Case & Deaton, growing mortality from overdose and alcohol misuse have been referred to as deaths of despair [8]. These deaths of despair are indicators of a breakdown in the social fabric of our communities [4, 9–10]. While demographic and clinical characteristics are important factors that influence these mortality rates, efforts to address factors related to chronic stress, such as social disconnectedness and economic strain, are often difficult, expensive, and take time to show change. Moreover, research suggests that economic distress is not distributed equally over time; some generations experience higher levels of economic disadvantage [11]. The impact of economic changes, such as standard of living, is unclear and often underutilized in this research [12–13]. However, exploring associations of these chronic risk factors, such as economic stress and social disconnectedness, within suicide and suicidal ideation (SI) might help improve suicide prevention interventions.

To date, research has identified a wide range of suicide risk factors across numerous domains, underscoring the complexity of addressing this public health problem [5]. Sociodemographic risk factors including being male or living in rural areas are associated with higher suicide rates [14–15]. In addition, mental health risk factors include a diagnosis of depression, anxiety, schizophrenia, or substance use disorder (SUD) [15–18]. Substance use, in particular the use of opioids, is also a significant risk factor for suicide attempt, especially among those with a co-occurring mental health diagnosis [19–20]. While other clinical factors are associated with suicide, such as suffering from non-cancer pain, additional life events are also significant risk factors connected to suicide, including adverse life events such as economic distress, legal problems, family disruption, or other types of social disconnectedness [17, 18, 21].

Despite the myriad of suicide risk factors that have been previously established in the literature, little is known about the impact of generational changes in financial stability in combination with other demographic, clinical, and social stressors. Such findings could provide insights to inform tailored upstream prevention efforts needed to curtail growing suicide rates, especially among known high-risk groups. The primary aim of this study is to examine sociodemographic, substance use, economic and social stressors associated with past year suicide within a representative sample of middle-aged adults in the United States. With SI being one of the strongest predictors of suicide mortality, it is essential to have a comprehensive understanding of factors associated with SI in order to help identify individuals at the highest risk before they attempt suicide [17, 22]. A secondary aim is to identify which of these factors were associated with single or repeated SI compared to no SI in this sample, thereby expanding the literature and current evidence.

## Methods

### Study sample

The study’s main goal was to compare White versus Black middle-aged adults (aged 40–60 years old). Participants were recruited using a stratified address-based sampling frame in order to identify households that included middle-aged adults (aged 40–60 years old). Using data from the 2020 American Community Survey, Census block groups were stratified by race/ethnicity (white, black), urbanicity, and educational achievement across eight demographic strata. Invitations were sent via postal mail to a total of 101,999 households sampled across the eight strata. Potential participants were randomly selected from among eligible middle-aged adults within responding households. Eligible respondents were offered $15 to participate and had the option to complete the survey either by mail or online. Initial surveys were completed between February and November 2022. The study’s final sample include 1,581 respondents who represented the adult population of persons aged 40–60 in the United States. Based on criteria from the American Association for Public Opinion Research, this represents a weighted response rate of 3.48% and a weighted completion rate of 58.11% [23]. After assessing for survey completion and accuracy, in particular for questions related to suicide, the study sample included 1,337 middle-aged adults. This study was approved by the institutional review board of the University of Illinois at Chicago (UIC) (protocol #2021 – 0485).

### Measures

SI and frequency were measured with two items from the Suicide Behaviors Questionnaire-Revised (SBQ-R) [24]. The primary outcome for the present study was the presence of thoughts of killing oneself in the past year (“How often have you thought of killing yourself in the past year?”; 1 = never, 2 = rarely (1 time), 3 = sometimes (2 times), 4 = often (3–4 times), 5 = very often (5 or more times)). If respondents failed to answer the question related to SI, they were removed from the sample for this analysis. A secondary outcome, SI frequency was categorized by number of times the respondent indicating they experienced SI in the past year. SI frequency was categorized in three mutually exclusive groups: no SI, single thought of SI, and repeated thoughts of SI.

We examined a comprehensive list of domains related to health and wellbeing, such as: sociodemographic, health status, and financial or social stressors. Sociodemographic characteristics included age (years), gender (cisgender male, cisgender female, transgender, non-binary, gender queer, or other gender identification), marital status (married or cohabitating, single, never married, separated, divorced, or widowed), race/ethnicity (Non-Hispanic White, Non-Hispanic Black, Hispanic or other ethnicity), educational attainment (GED or less, high school diploma, or some education post-high school), metropolitan statistical area (urban/suburban or rural), health insurance status (no health insurance, private health insurance or public health insurance). Health status included self-reported health (excellent, very good, good, fair, or poor).

Perceptions of change in economic status as an indicator of economic hardship was assessed by asking “Think about whomever was mainly responsible for raising you when they were the age you are now. Do you think your own standard of living now is much better, somewhat better, about the same, somewhat worse, or much worse than theirs was?” Responses were collapsed into the following groups: better off, about the same, or worse off than their parents/caregivers. As another way to explore the impact of where the respondents live, we examined Social vulnerability index (SVI). This is a community level measure that is comprised of factors related to poverty, lack of access to transportation, and housing environment. SVI was developed by the Centers for Disease Control and Prevention (CDC) and has been found to be directly related to several health outcomes including suicide [25–27].

Social support was measured with a modified version of the Social Support Network Inventory (SSNI), as this tool has been validated and studies demonstrate strong psychometric properties supporting the use as a proxy for social support, which is well known to mediate service mental health issues [28]. A score representing total social support was calculated based on responses related to levels of support for life issues and stressful experiences. The SSNI captures the amount of support an individual has in relation to intimacy, practical help, emotional support and reassurance of self-worth from the most important person to the respondent in each of four categories: partner/spouse, co-worker, friend outside of work, and relative. Support in each area and from each source are assessed on a 7-point Likert-type scale ranging from 1 to 7, with lower scores representing less support and higher scores indicating higher levels of social support. Ratings are summed for each type of support and then averaged over the number of sources of support listed by a respondent, to create a total support score.

Recent substance use was assessed for alcohol, marijuana, opiates, or other illicit substances. Due to small sample sizes, other substances were grouped together and included heroin, methamphetamine, crack, hallucinogens, and inhalants. Respondents who reported using these substances in the past month were further categorized depending on number of substances. Dummy coded variables were created for those who reported any substance use or concurrent substance use. Concurrent substance use was defined as using two or more substances in the past month.

### Statistical analysis

Data for this study was analyzed using SAS v9.4 (Cary, North Carolina). Descriptive analysis for all potential predictors were completed using cross tabulations and alpha was set to 0.05 for all tests unless otherwise specified. Bivariate analyses were conducted using chi-square test of differences, for all variables in the dataset known to be linked to suicide or mental health distress (Table 1).

Using multivariable logistic regression, the aORs for past year SI were calculated for sociodemographic, substance use, economic and social support variables. Multivariable logistic regression was used to provide a more direct interpretation for the relationship between the predictors and SI. A subsequent multinomial logistic regression was performed to identify factors associated with single or repeated SI compared to those with no past year SI among the study population.

Assumptions were tested by assessing multicollinearity among predictors using a variance inflation factor (VIF) with a critical value greater than 10.0. Goodness of fit indices such as the Type 3 Analysis of Effects and Wald chi-square test for maximum likelihood estimates were examined for all predictors.

Adjusted odds ratios (aOR) with 95% Confidence Intervals (CIs) were calculated for each of the significant predictors in the final model. Odds ratios indicate factors significantly associated with increased odds of reporting SI in the past year, adjusted for all potential confounders. Similarly, using a multinomial logistic regression model can produce aORs and corresponding 95% CIs for those significant factors associated with SI one vs. multiple times in the past year.

## Results

Among the study sample of 1,337 middle-aged adults, 10.5% (n = 140) reported past year SI. Those who reported SI were more often cisgender male (66.4%, p < 0.001) and were single, separated, divorced, or widowed (64.3%, p = 0.03). Similarly, those who were Non-Hispanic white (p < 0.001) or with a household income under $30,000 (p = 0.02) more often reported SI. Those who were not employed (p < 0.001) or reported worsening standard of living compared to their parents/caregivers (p < 0.001) more often reported past year SI. Moreover, those living in areas with lower SVI scores more often reported past year SI (median = 0.78, interquartile range [IQR]: 0.60–0.91). A larger proportion of those with past year SI reported fair or poor health (p < 0.001) or reported recent substance use (p = 0.03), in particular concurrent substance use (p < 0.001). Alcohol and marijuana use were the substances most often reported by the study sample, with higher rates of use and co-use among those with past year SI. In addition, prescription opiate use including heroin use was more often reported among those with past year SI. (Table 1) Moreover, 60.0% (n = 84) of those with past year SI reported past year SI two or more times, or multiple SI.

Table 2 shows results of a multivariable logistic regression analysis by SI status in the past year after adjusting for other confounding factors. Those who were gender minorities (e.g., transgender, non-binary, gender queer, or other gender identity) had more than 7 times the odds of SI compared to cisgender females. Those who engaged in concurrent substance use in the past month had over three times the odds of SI. Those experiencing economic stressors, such as unemployment, had nearly twice the odds of experiencing SI. Similarly, those who reported a worse standard of living than their parents/caregivers had nearly three times the odds of past year SI compared to those who did not report any change between generations. Social support, as measured by SSNI, was a significant protective factor for SI. Those with higher social support network inventory scores had lower odds of past year SI. In particular, for each point higher the respondent scored in the SSNI, they were 31% less likely to report either SI over the past year. In addition to higher levels of support, those who were Non-Hispanic Black were approximately 50% less likely to report repeated SI.

Table 3 shows results from a multinomial regression analysis by SI status: single ideation, multiple ideation, compared to no SI in the past year (reference group). The most significant predictor of single or multiple SI was concurrent substance use in the past month. Those who reported the use of multiple substances in the past month had more than three times the odds of SI. Those who were unemployed had roughly twice the odds of past year SI. Interestingly, those who reported a worse standard of living than their parents/caregivers had nearly 3 times the odds of multiple SI in the past year. Similar to results in Table 2, higher scores for the SSNI were protective for both single and multiple SI among middle-aged adults.

## Discussion

The findings of this cross-sectional study in a large sample of middle-aged adults document the impact of social and economic stressors and highlight disparities related to SI. These results are consistent with research on gender minorities and demonstrate the elevated risk of suicide among this often-underrepresented population [29–30]. These significant associations between gender identity, concurrent substance use, social support, and economic hardship in relation to suicide risk are critical when developing both public and clinical interventions. The present study adds to and extends the literature in two ways. First, to the best of our knowledge, it is the first to report data on a national sample of middle-aged adults and examine the relationship between perceived intergenerational changes in economic status and SI. Second, it is one of the first to examine the relationship between these generational changes, social support, and concurrent substance use. Although some research does suggest that those who identify as transgender or non-binary more often face mental health and substance use issues, many of these studies are among younger adults or the Veteran population [29, 31–35].

One major finding of this study is the factors associated with multiple SIs within a year, which included utilization of multiple substances in the past month, unemployment, and self-perception that the participant is worse off than one’s parents or caregivers. SI that is more frequent has been associated with suicide intent in veterans and suicide attempts in adult outpatients, these factors should be particularly considered for public health interventions [36–37]. For example, there is evidence to suggest that SI can be decreased in individuals with substance use disorders who undergo treatment for substance use disorder [38]. More specific interventions for those at higher risk for SI should be developed and researched to determine their effectiveness.

### Limitations

However, this study is not without limitations. First, all measures, including those for mental health, economic and social stressors, are from self-reported data. Second, the ability to assess frequency and time of concurrent substance use was limited based on survey questions. Additional research on contextual elements that influence concurrent substance use is needed. Third, this survey largely surveyed middle-aged adults who identified as Non-Hispanic White or Black, which limits the generalizability of results to other racial groups. The small sample sizes for middle-aged adults who reported being a minority group member could impact statistical power needed to detect differences and ascertain potential racial disparities that exist. In addition, future work should explore the intersection of mental health disparities by gender and race/ethnicity. Moreover, these results are based on cross-sectional data and cannot assess causality. Additional research is needed to explore the intersection of suicide and these various risk and protective factors over time among middle-aged adults.

## Conclusions

Overall, these results suggest the need for a more comprehensive approach to improve suicide rates among middle-aged adults. In particular, improving social and economic support systems, especially among those who are a gender minority would be valuable to reduce SI. Public health interventions that reduce stigma and provide psychosocial support among those at highest risk could also result in an improvement of mental health distress. In addition, interventions that improve the sense of belonging and acceptance in the community could be another way to mitigate potential suicide risk among this population. With respect to concurrent substance use, improving access to mental health treatment and harm reduction resources could improve multiple health outcomes, including suicide. Future work should identify interventions and policies that reduce suicide risk, especially among those experiencing higher levels of social or economic distress.

## Figures and Tables

**Table 1:** Characteristics of middle-aged adults by past year suicide ideation status

	Overall		No suicide ideation (n = 1,197)		Suicide ideation (n = 140)		p-Value
Demographic	Median	IQR ^a^	Median	IQR ^a^	Median	IQR ^a^	
*Age*	50	44–56	50	44–56	50	43–56	0.69
*Social vulnerability index (SVI)*	0.80	0.58–0.92	0.80	0.58–0.92	0.78	0.60–0.91	< 0.001*
*Social support network inventory score*	4.75	3.67–5.71	3.75	5.77	4.07	3.00–5.00	< 0.001*
	**N**	**%**	**n**	**%**	**n**	**%**	
**Gender**							< 0.001*
*Cisgender male*	849	63.50	756	63.16	93	66.43	
*Cisgender female*	471	35.23	429	35.84	42	30.00	
*Transgender, non-binary etc.*	11	0.82	6	0.50	5	3.57	
**Marital Status**							0.03*
*Single, never married, separated, divorced, widowed*	727	54.38	637	53.22	90	64.29	
*Married or cohabitating*	596	44.58	546	45.61	50	35.71	
**Race/ethnicity**							< 0.001*
*Non-Hispanic White*	653	48.84	570	47.62	83	59.29	
*Non-Hispanic Black*	435	32.54	405	33.83	30	21.43	
*Hispanic or other race*	249	18.62	222	18.55	27	19.29	
**Household income**							0.02*
*Under $30,000*	574	42.93	498	41.60	76	54.29	
*$30,001 to $60,000*	319	23.86	288	24.06	31	22.14	
*$60,000-$90,000*	166	12.42	156	13.03	10	7.14	
*Over $90,000*	239	17.88	217	18.13	22	15.71	
**Educational attainment**							0..37
*High school diploma, GED, or less*	468	35.00	423	35.34	45	32.14	
*Some education post-high school*	858	64.17	763	63.74	95	67.86	
**Employment status**							< 0.001*
*Not employed*	491	36.72	419	35.00	72	51.43	
*Employed*	807	60.36	739	61.74	68	48.57	
**Rurality**							0.65
*Urban/suburban*	625	46.75	557	46.53	68	48.57	
*Rural*	712	53.25	640	53.47	72	51.43	
**Health insurance status**							0.07
*No health insurance*	217	16.23	200	16.71	17	12.14	
*Private health insurance*	500	37.40	455	38.01	45	32.14	
*Public health insurance*	483	36.13	417	34.84	66	47.14	
*Public and private insurance*	134	10.02	122	10.19	12	8.57	
**Health status**							< 0.001*
*Good, very good, or excellent*	968	72.40	892	74.52	76	54.29	
*Fair or poor*	366	27.37	302	25.23	64	45.71	
**Change in standard of living**							< 0.001*
*Worse*	227	16.98	183	15.29	44	31.43	
*About the same*	282	21.09	264	22.06	18	12.86	
*Better*	748	55.95	683	57.06	65	46.43	
**Substance use**							
*Binge drank in past year*	435	32.54	388	32.41	47	33.57	0.80
**Any substance use in past month**							0.003*
*No*	653	48.84	601	50.21	52	37.14	
*Yes*	684	51.16	596	49.79	88	62.86	
**Concurrent use of substances in past month**							< 0.001*
*No*	1,203	89.98	1,098	91.73	105	75.00	
*Yes*	134	10.02	99	8.27	35	25.00	
**Type of substance use in past month**							
*Alcohol use*	597	44.65	523	43.69	74	52.86	0.10
*Marijuana use*	183	13.69	143	11.95	40	28.57	< 0.001*
*Opiate use*	39	2.92	29	2.42	10	7.14	0.007*
*Other substance use*	20	1.50	15	1.25	5	3.57	0.03*

a Interquartile range (IQR)

* Indicates statistical significance (p < 0.05).

**Table 2:** Factors associated with past year suicide ideation among middle-aged adults in the United States

Variable	aOR ^a^	95% CI ^b^
**Gender**		
*Cisgender male*	1.47	0.96–2.26
*Cisgender female*	Ref	-
*Transgender, non-binary, etc.*	7.06*	1.91–26.04
**Race**		
*Non-Hispanic White*	1.02	0.61–1.70
*Non-Hispanic Black*	0.52*	0.28–0.95
*Hispanic or other race*	Ref	-
**Change in standard of living**		
*Worse*	2.62*	1.41–4.87
*About the same*	Ref	-
*Better*	1.67	0.94–2.99
**Employment status**		
*Employed*	Ref	-
*Not employed*	1.80*	1.21–2.68
**Concurrent use**		
*No concurrent use*	Ref	-
*Concurrent use*	3.29*	2.02–5.36
**Social support network inventory score**	0.69*	0.60–0.79

a Adjusted Odds Ratio (aOR)

b 95% Confidence Interval (CI)

* Indicates statistical significance (p < 0.05).

**Table 3:** Factors associated with single or repeated suicide ideation among middle-aged adultsin the United States

	Single ideation		Multiple ideation	
Variable	aOR ^a^	95% CI ^b^	aOR ^a^	95% CI ^b^
**Change in standard of living**				
*Worse*	1.58	0.68–3.66	2.99*	1.39–6.41
*About the same*	Ref	-	Ref	-
*Better*	1.45	0.70–3.02	1.50	0.72–3.12
**Employment status**				
*Employed*	Ref	-	Ref	-
*Not employed*	2.08*	1.22–3.56	1.79*	1.10–2.93
**Concurrent substance use**				
*No concurrent use*	Ref	-	Ref	-
*Concurrent use*	3.13*	1.62–6.03	3.17*	1.76–5.70
**Social support network inventory score**	0.75*	0.62–0.90	0.65*	0.55–0.78

a Adjusted Odds Ratio (aOR)

b 95% Confidence Interval (CI)

* Indicates statistical significance (p < 0.05).

## Data Availability

Data for participants who agreed to data sharing will be made available through the National Institute on Alcohol Abuse and Alcoholism Data Archive.
